# Molecular and biochemical characterization of wheat resistance to yellow rust (*Puccinia striiformis* f. Sp. tritici) using SSR markers and antioxidant profiles

**DOI:** 10.1186/s12870-025-07380-2

**Published:** 2025-09-26

**Authors:** Amira M. Ghanaim, Marwa M. Ghonaim, Khaled I. Gad, Heba I. Mohamed, Ahmed A. A. Omran

**Affiliations:** 1 Department of Biological and Geological Sciences, Faculty of Education, Ain Shams University, Cairo, 11341, Egypt; 2 Cell Study Research Department, Field Crops Research Institute, Agriculture Research Center, Giza, Egypt; 3 Department of Wheat Research, Institute of Research Field Crops, Agricultural Research Centre (ARC), Giza, Egypt

**Keywords:** Antioxidant activity, Flavonoids, Phenolic, SSR, Yellow rust, Wheat

## Abstract

**Background:**

One of the most damaging foliar diseases of wheat in Egypt and around the world is yellow rust, often known as stripe rust, which is caused by *Puccinia striiformis* f. sp. *tritici*. This study aims to identify wheat cultivars resistant to yellow rust and to detect molecular markers linked to resistance. Two Egyptian bread wheat cultivars, *Yr10* and *Yr18* (highly resistant), and Gemmiza 11 and Shandweel 1 (susceptible), were evaluated. Four crosses and subsequent backcrosses between resistant and susceptible bread wheat genotypes were obtained. The phenolic content and antioxidant activity of the cultivars and their crosses under disease conditions were also measured for potential use in wheat breeding programs.

**Results:**

The results showed that Gemmiza 11 and Shandweel 1 were highly susceptible and susceptible wheat cultivars, respectively. On the other hand, the two Yr monogenic lines (*Yr10* and *Yr18*) showed high resistance against stripe rust. In addition, the cross between the susceptible cultivars and the resistant monogenic lines exhibited immunity and high resistance to *Puccinia striiformis* (*Pst*). The highest phenolic and flavonoid contents were recorded in the immune cultivars. In addition, all immune wheat cultivars exhibited higher levels of superoxide anion radical, hydroxyl radical, and nitric oxide radical scavenging activity. Out of thirteen SSR markers tested, seven were found to be associated with resistance to yellow rust.

**Conclusion:**

These markers may serve as the basis for marker-assisted selection programs aimed at enhancing wheat resistance to yellow rust.

## Introduction

Wheat (*Triticum aestivum* L.) is one of the most important cereal crops worldwide and a staple in the Egyptian diet [1]. In Egypt, however, there is a 50% gap between production and consumption, leading to an insufficient domestic supply [2]. Wheat productivity is affected by several biotic and abiotic stresses, among which *Puccinia striiformis* f. sp. tritici (*Pst*), the causal agent of yellow rust, is the most destructive. Because most widely cultivated wheat varieties are susceptible, yellow rust poses a serious threat in northern Egypt [3]. Disease incidence is often higher with late sowing, when environmental conditions favor rust development [4]. Highly susceptible cultivars suffer significant yield losses, especially with the recent emergence of new virulent races of the pathogen [5]. The danger of stripe rust lies in its high mutation capacity, short generation turnover, and ability to spread over long distances [6].

Furthermore, the limited genetic diversity among Egyptian wheat cultivars poses a serious challenge, as it could increase the virulence of stripe rust and significantly reduce wheat productivity and yield in Egypt. The most often used bread wheat cultivars in Egypt are Shadweel 1 and Gemmeiza 11, particularly for small farms. Farmers favor those varieties because of their superior bread-making qualities [7]. Unfortunately, Gemmeiza 11 and Shandweel 1 are highly susceptible to stripe rust disease. Without timely detection and appropriate chemical management, substantial yield losses occur each year. The most effective and sustainable strategy to reduce these losses is the introgression of yellow rust resistance genes into these cultivars [8]. The most efficient resistance genes against the predominant *Pst* races in Egypt are *Yr5*, *Yr10*,* Yr15*, and *YrSp* [8, 9]. Traditional methods of controlling *Pst* disease have relied on the use of chemical pesticides, which can have adverse environmental impacts and contribute to the development of pesticide resistance [10]. Fungicides can be used to manage stripe rust; however, developing resistant wheat genotypes is a more practical, affordable, and environmentally sustainable approach [10]. Since chemical control raises concerns about food safety, environmental impact, and pathogen resistance, breeding resistant variants represents the most promising long-term strategy [11].

Identification of resistance genes effective under the target environment is a critical step in breeding for stripe rust resistance. Such genes provide the foundation for resistance breeding and marker-assisted selection [12]. Molecular markers serve as powerful tools for assessing genetic relationships within and among genotypes and for evaluating genetic diversity. The presence of resistance genes can be detected through wheat genotyping using different marker systems, including Simple Sequence Repeats (SSR) and Diversity Arrays Technology (DArT) [13].

A wide range of molecular markers has been developed to assess genetic variation and diversity, such as Restriction Fragment Length Polymorphism (RFLP), Random Amplified Polymorphic DNA (RAPD), Inter-Simple Sequence Repeats (ISSR), Amplified Fragment Length Polymorphism (AFLP), retrotransposon-based markers, and Single Nucleotide Polymorphisms (SNPs) [14–19]. Modern wheat breeding has greatly benefited from molecular technologies developed over the past few decades. The identification of molecular markers linked to resistance genes, and their subsequent use in breeding programs, facilitates more efficient selection [20, 21]. Among these, SSR markers are the most widely used in wheat disease resistance gene mapping and genetic improvement, as they are highly polymorphic, reproducible, and cost-effective [21].

When wheat plants are challenged by *Pst*, a complex biochemical defense network is activated to restrict pathogen entry, colonization, and spread. These mechanisms are largely mediated through the regulation of reactive oxygen species (ROS), antioxidant systems, and secondary metabolites [22]. Upon pathogen recognition, wheat cells produce a rapid and localized accumulation of ROS such as superoxide anion (O₂⁻), hydrogen peroxide (H₂O₂), and hydroxyl radicals (•OH). This oxidative burst serves a dual role: directly damaging fungal structures and functioning as a signaling molecule to activate downstream defense pathways [23]. Excessive ROS can, however, be harmful to host cells, making fine-tuned regulation critical [18].

Toxic ROS, particularly hydroxyl radicals (•OH), can damage proteins, lipids, and nucleic acids. Since cells lack enzymatic mechanisms to eliminate •OH, its excessive accumulation can cause severe cellular injury or death. Therefore, the ability to scavenge hydroxyl radicals represents a vital antioxidant function and a strong predictor of overall antioxidant potential [24, 25]. Plants have evolved both enzymatic and non-enzymatic antioxidant systems to mitigate the harmful effects of ROS generated during host–pathogen interactions [26]. Non-enzymatic antioxidants include carotenoids, flavonoids, and phenolic compounds. Flavonoids, in particular, are polyphenolic molecules with strong antioxidant activity; by donating electrons to radicals, they form less reactive intermediates and help maintain redox balance [27]. In wheat–*Puccinia striiformis* interactions, resistant cultivars often accumulate higher levels of phenolics, flavonoids, and carotenoids at infection sites, which strengthen cell walls, limit pathogen spread, and reduce oxidative damage. This biochemical reinforcement complements enzymatic defenses, highlighting the central role of antioxidant systems in stripe rust resistance [24–25; 27].

Although many studies separately address molecular resistance genes or antioxidant responses, few have combined molecular marker analysis with biochemical profiling to better understand wheat resistance to yellow rust. This integrative approach can provide novel insights into both genetic and physiological resistance mechanisms. The current study aimed (1) to assess the effectiveness of introducing the two stripe rust resistance genes, *Yr10* and *Yr18*, in enhancing the resistance of the two rust-sensitive bread wheat cultivars, Shandweel 1 and Gemmeiza 11; (2) to evaluate the reductions in grain production caused by stripe rust infection in the wheat cultivars that were examined; and (3) to study the role of different free radical scavenging activities and antioxidants in imparting tolerance to wheat cultivars against yellow rust.

## Materials and methods

### Experimental site

This investigation was carried out under field conditions at the experimental farm of Sakha Agricultural Research Station, Kafr El-Sheikh Governorate, northern Egypt (31° 5’ 12” N; 30°56’ 49” E).

### Plant materials

The Wheat Research Department of the Field Crops Research Institute, Agricultural Research Centre (ARC), Egypt, produced two Egyptian bread wheat cultivars, Shandweel 1 and Gemmeiza 11 (Table 1). The International Centre of Agricultural Research in Dry Areas (ICARDA) gave two monogenic lines (*Yr10* and *Yr18*) for stripe rust.x`x

**Table 1:** List of the local bread wheat genotypes that were used, pedigree and year of release

Genotype	Designation	Year of release
Gemmeiza 11	B0W"S”/KVZ"S”//7 C/SERI82/3/GIZA168/SAKHA61.GGM7892-2GM-1GM-2GM-1GM-0GM.	2011
Shandaweel 1	SITE/MO/4/NAC//*PVN/3/MiRLO	2012

###  Crossing and field evaluation

In order to synchronize flowering, the two Egyptian bread wheat cultivars and the two Yr monogenic lines were sown at three planting dates during the 2018/2019 season. Each parent was planted twice, with a length of 2.0 m between each planting date. To create F1 seeds, the two wheat cultivars (stripe rust susceptible parents) were crossed with the two resistant parents that had the genes *Yr10* and *Yr18.*

In the 2019/2020 season, the F1 seeds of each cross were sown in rows of 2.0 m long and 30 cm apart and spaced widely at 30 cm apart in order to allow for the production of backcrosses in the 2020/2021 season. All materials were collected and sown in a field experiment and were arranged in a randomized complete block design with three replications. The experimental materials included four parents (Gemmiza 11, Shandweel 1, *Yr18*, and *Yr10*), four crosses (*Yr18* × Shandweel 1, *Yr10* × Shandweel 1, *Yr10* × Gemmiza 11, and *Yr18* × Gemmiza 11), and four backcrosses (BC1, BC2, BC3, and BC4). Each entry was planted in rows 2.0 m long, with 30 cm between rows and 20 cm between plants. Each plot consisted of 36 rows.

To achieve a consistent distribution of *P. striiformis* inoculum, the highly susceptible spreader wheat cultivar (Morocco) was planted around the experimental area. Using a modified Cobb’s scale, the reactions of every plant to stripe rust were noted when the plant reached maturity [28].

### Disease assessment

Spreader row plant inoculation was done using Tervet and Cassel [29] approach during the wheat booting stage. Using a modified Cobb’s scale [30], the reactions of all the investigated wheat genotypes to the *P. striiformis* pathotypes were noted at the adult plant stage. As indicated in Table 2, infection types were ranked in the adult stage using Leath and Heun [31] methodology.

**Table 2:** Infection types of inoculated wheat at adult stage

Infection type	Host response	Symptoms
0	Immune	No visible signs or symptoms
1	Highly resistant	Small flecks only
2	Resistant	Chlorosis flecks evident
3	Moderately resistant	Large flecks with chlorosis and necrosis
4	Intermediate	Mycelium and conidia detectable
5	Moderately susceptible	Small to moderate-sized pustules and conidia present
6	Moderately susceptible	Predominance of moderate-sized pustules and conidia present
7	Susceptible	At the least 50% of the large pustules and conidia are visual
8	Susceptible	75 to 80% of the leaf segment were covered with large pustules and conidia
9	Highly susceptible	100% of the leaf segment curved with large pustules and conidia

### Biochemical analysis

#### Extraction and Estimation of free radical scavenging activities

Leaf samples (2 g) from wheat cultivars and their crosses were extracted with 5 mL of methanol by incubating the test tubes in a water bath at 70 °C for one hour. The extracts were then filtered through Whatman No. 1 filter paper, adjusted to a final volume of 5 mL, and subsequently used to determine total phenolic content, flavonoid content, and free radical scavenging activity in the different wheat cultivars.

#### Estimation of non-enzymatic antioxidants

The Folin–Ciocalteu technique was adjusted to measure total phenolic concentrations. After mixing 100 µL of methanol extract with 500 µL of 2 N Folin–Ciocalteu reagent, 1.5 mL of 1.89 M anhydrous sodium carbonate solution, and 7.9 mL of distilled water, the mixture was left in the dark for 120 min. After that, it was centrifuged for five Minutes at 4000 ×g, and a spectrophotometer (Shimadzu 150 UV-1800, Kyoto, Japan) was used to measure the absorbance at 760 nm by Swain and Hills [32]. Total phenolic content was quantified and expressed in terms of gallic acid equivalents (GAE).

To measure the flavonoid concentration, the filtrate was also combined with 4 ml of distilled water. Then, 0.3 ml 5% sodium nitrite was added. After 5 min, 0.3 ml of 10% aluminium chloride was added. In 6 min, 2 ml of 1 M sodium hydroxide was added to the mixture. Immediately, the mixture was diluted by the addition of 3.3 ml distilled water and mixed thoroughly. The absorbance was determined at 510 nm versus a blank [33]. Catechin was used as a standard for the calibration curve. Total flavonoid content of the extract was expressed as mg catechin equivalents per gram of sample (mg/g).

#### Estimation of free radical scavenging activities

The free radical scavenging activity was assessed using the 2,2-diphenyl-1-picrylhydrazyl (DPPH) assay. A 0.5 mL aliquot of the methanolic extract was mixed with DPPH solution, and the decrease in absorbance was recorded at 515 nm against a reagent blank, following the method of Bolis [34]. In addition, the ferric reducing antioxidant power (FRAP) assay, as described by Benzie and Strain [35], was used to evaluate antioxidant potential. In this assay, antioxidants in the extract reduce Fe³⁺ to Fe²⁺ in the presence of 2,4,6-tris(2-pyridyl)-s-triazine (TPTZ), producing a blue-colored Fe²⁺–TPTZ complex. The absorbance of this complex was measured at 593 nm, and FRAP values were calculated using FeSO₄·7 H₂O as the standard.

The antioxidant capacity of wheat extracts against ABTS radicals was determined using a modified method of Rice-Evans and Miller [36]. Briefly, 100 µL of the methanolic extract was mixed with 2 mL of ABTS solution and incubated at 30 °C for 6 min. Following incubation, the decrease in absorbance was measured at 734 nm using a spectrophotometer.

Total reducing power was determined according to Oyaizu [37] with slight modifications. The reaction mixture consisted of 200 mM sodium phosphate buffer (pH 6.6), potassium ferricyanide (1%, w/v), trichloroacetic acid (TCA), and FeCl₃ (0.1%, w/v). The intensity of the resulting green coloration was measured spectrophotometrically at 700 nm against a reagent blank. Superoxide radical (O₂•⁻) scavenging activity was assessed using the standard method of Marklund and Marklund [38], based on the inhibition of pyrogallol autoxidation. Absorbance was recorded at 420 nm to quantify the scavenging potential.

Hydroxyl radical scavenging activity was assessed based on the Fenton-type reaction, which generates hydroxyl radicals. The reaction mixture contained 1,10-phenanthroline, FeSO₄, methanolic extract, sodium phosphate buffer, and 0.02% hydrogen peroxide. The mixture was incubated at 37 °C for 1 h, and absorbance was recorded at 536 nm against a reagent blank, following a modified protocol of Min et al. [39]. Nitric oxide (NO•) scavenging activity was determined using the method of Marcocci et al. [40]. In this assay, sodium nitroprusside generates nitric oxide, which reacts with oxygen to form nitrite ions. These nitrite ions were quantified using the Griess reagent, and absorbance was measured spectrophotometrically to estimate NO• scavenging potential.

### Molecular analyses

Following the CTAB (Cetyl Tri-methyl Ammonium Bromide) extraction procedure, genomic DNA was extracted from the leaves of the investigated wheat genotypes and the positive control, NIL Jupateco 73 R, as demonstrated by Chen et al. [41]. Young leaf tissues (approximately 100 mg) were ground in liquid nitrogen and suspended in preheated CTAB extraction buffer, followed by incubation at 65 °C. The lysate was treated with chloroform: isoamyl alcohol (24:1 v/v) to remove proteins and other contaminants, and the aqueous phase was collected. DNA was precipitated with cold isopropanol, washed with 70% ethanol, air-dried, and finally resuspended in TE buffer (10 mM Tris-HCl, 1 mM EDTA, pH 8.0). The quality and concentration of the extracted DNA were checked using agarose gel electrophoresis and spectrophotometric measurement. Table 3 displays the several SSR primers that were used for the 10 µL PCR amplification. In an Eppendorf Mastercycler^®^ Gradient, PCR was run for three Minutes at 94°C. This was followed by 45 cycles of 15 s each at 94 °C, 58 °C, and 72 °C, and a final extension step of 10 min at 72 °C. After staining with ethidium bromide (500 µL L⁻¹), the PCR products were resolved by electrophoresis in 1.2% agarose gel and seen under ultraviolet light.

**Table 3:** SSR primers for yellow rust resistance genes *Yr10* and *Yr18*

Primer Name	Forward primer	Reveres primer	Fragment size	Tm °C
XpSp3000	5’GCAGACCTGTGTCATTGGTC3’	5’GATATAGTGGCAGCAGGATACG3’	286	55
E1	5’CTTGCTGGCGACCTGCTTA 3’	5’TGTTTCGCTCCACGCTGACT 3’	754	55
E2 Upstream	5’TGGTAGTAGAGTAATCGCAACA3’	5’TCTTCAGATTTGGAGGTAGG3’	377	60 or 55
E2A Downstream	5’TGGAAATGGATAGGCGAAGG 3’	5’AAATCAATGAAGCCGCAACC 3’	872	55
Yr10	5’TCAAGGAGGTCAGTGACAG3’	5’TCAGGGAGGTGTAGCCTAAT 3’		
Yr10 F/Yr10	5’TCAAAGACATCAAGAGCCGC 3’	5’TGGCCTACATGAACTCTGGAT 3’	+ 543	64
Yr10F/Yr10R1	5’TTGGAATTGGCGACAAGCGT 3’	5’GTGATGATTACCCACTTCCTC 3’	+ 755	64
GWM295	5’ GTGAAGCAGACCCACAACAC 3’’	5’ GACGGCTGCGACGTAGAG 3’		60
L34DINT9F L34PLUSR	5’TTGATGAAACCAGTTTTTTTTCTA3’	5’GCCATTTAACATAATCATGATGGA3’	+ 517	58
L34DINT9F L34MINUSR	5’TTGATGAAACCAGTTTTTTTTCTA 3’	5’TATGCCATTTAACATAATCATGAA 3’	−523	58
CsLV34	5’GTTGGTTAAGACTGGTGATGG3’	5’TGCTTGCTATTGCTGAATAGT 3’	+ 150 −229	58
CslvLr	5’GTTGGTTAAGACTGGTGATGG3’	5’TGCTTGCTATTGCTGAATAGT 3’		
Lr34Delta	5’CATTATTTTTTTCCATCATG 3’	5’ATGAAGCAATAAATCGATG 3’		

### Data analysis

The band profiles of SSR primers were scored based on the presence (1) or absence (0) of distinct bands. Using thirteen SSR primer banding patterns, the degree of genetic relatedness among the genotypes in this study was determined. The algorithms from the NTSYS-PC (Numerical Taxonomy and Multivariate Analysis System), version 2.1 (Applied Biostatistics) program were utilized to compute the similarity coefficients: the Unweighted Pair Group Method with Arithmetic Averages (UPGMA) and SAHN (Sequential, Agglomerative, Hierarchical, and Nested Clustering) [42]. Furthermore, PAST software 4.02 (https://www.nhm.uio.no/English/research/infrastructure/past/) was used to generate a principal component analysis (PCA) based on the SSR data matrix. ClustVis, an online tool for visualizing multivariate data clustering (https://biit.cs.ut.ee/clustvis/), was used to construct heat maps [43].

### Statistical analysis

Using Duncan’s multiple range test [44], the significance of the mean of three replications in a randomized complete block design was assessed for all quantifiable parameters according to the protocol described by Gomez and Gomez [45].

## Results

### Infection types and disease severity

**Fig. 1:**
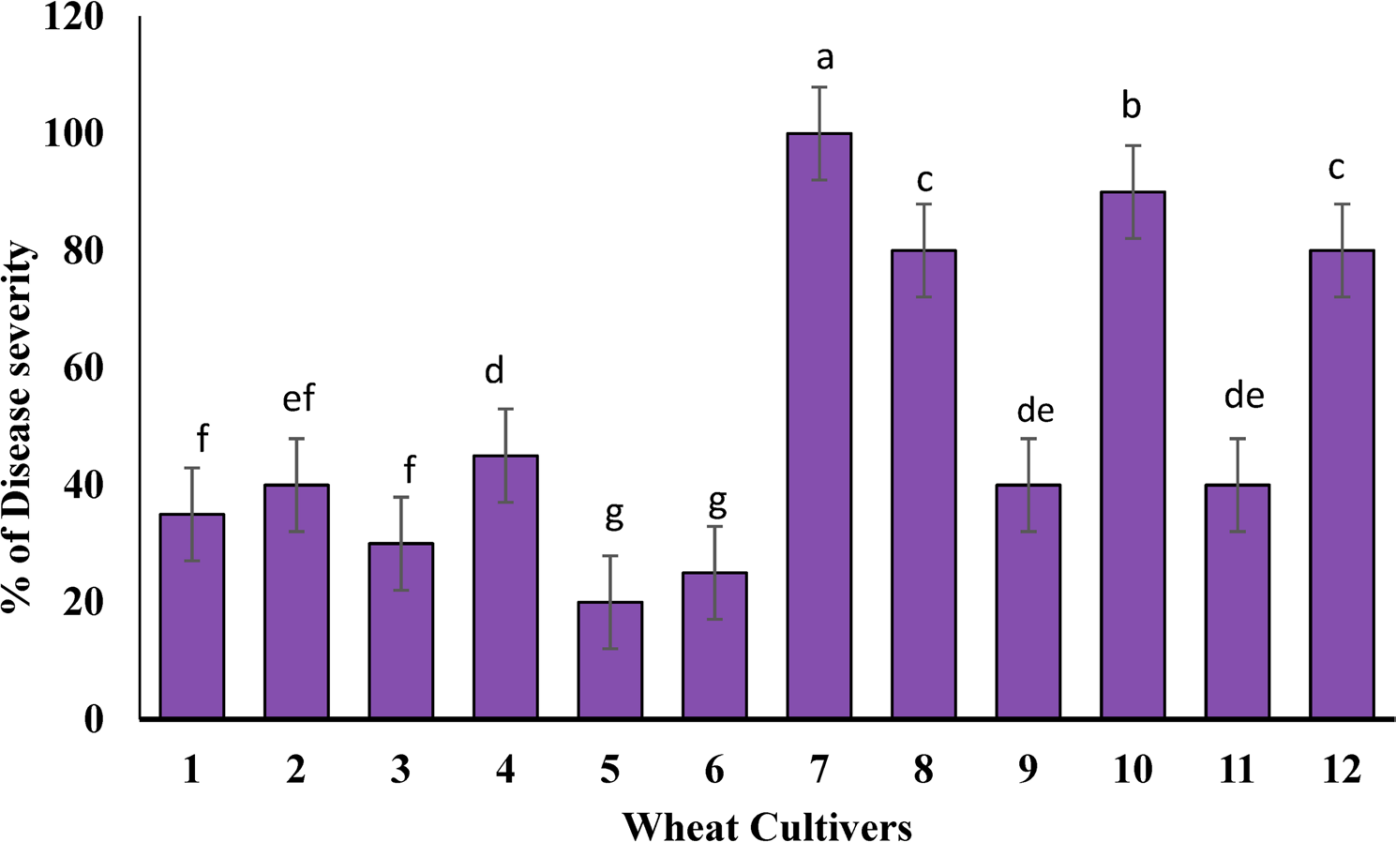
the disease severity of yellow rust in wheat cultivars and crosses. Mean values of three replicates and standard deviation (± SD) in each bar followed by a different lowercase letter are significantly different according to Duncan’s multiple range tests at *p* ≤ 0.05. 1: *Yr18* × Shandweel 1, 2: *Yr10* × Gemmiza 11, 3: *Yr10* × Shandweel 1, 4: *Yr18* × Gemmiza 11, 5: *Yr10*, 6: *Yr18*, 7: Gemmiza 11, 8: Shandweel 1, 9: BC1, 10: BC2, 11: BC3, 12 : BC4

The infection types and disease severity of wheat stripe rust caused by *P. striiformis* showed significant differences among all examined genotypes and crosses, as presented in Table 3; Fig. 1. The susceptible cultivars Gemmiza 11 and Shandweel 1 exhibited high susceptibility, with disease severity reaching approximately 100% and 80%, respectively. In contrast, the two Yr monogenic lines (*Yr10* and *Yr18*) displayed high resistance to stripe rust, with lower disease severity values of about 20% and 25%, respectively. Crosses between the susceptible cultivars and the resistant monogenic lines exhibited immune to highly resistant responses against *Pst*. The backcrosses varied in their reactions: BC1 and BC3 showed high resistance with disease severities of about 40% and 45%, respectively, whereas BC2 and BC4 were susceptible, exhibiting higher disease severities of about 90% and 80% (Fig. 1).

### Evaluation of morphological and yield attributes

The results in Table 4 revealed clear differences between susceptible and resistant entries. Overall, the susceptible cultivars (Gemmiza 11 and Shandweel 1) along with BC2 and BC4 exhibited significantly lower values for most morphological and yield traits compared to the other crosses and backcrosses. In contrast, the two Yr monogenic lines (Yr10 and Yr18), their crosses, and the backcrosses BC1 and BC3 outperformed the susceptible entries, showing the highest values for most traits.

**Table 4:** Effect of yellow rust on morphological and yield attributes of wheat cultivars and crosses

Cultivars and crosses	Plant height (cm)	No. of tillers	No. of green spike	1000 weight seeds (g)	Grain yield (g)	Infection type
*Yr18* × Shandweel 1	122.1 ± 4.0^b^	26.2 ± 1.0^b^	44.6 ± 1.0^de^	42.13 ± 1.0^d^	38.11 ± 1.0^b^	Immune
*Yr10* × Gemmiza 11	117.1 ± 3.0^c^	23.4 ± 1.0^c^	51.6 ± 2.0^c^	44.40 ± 1.0^c^	35.25 ± 1.0^c^	Highly resistant
*Yr10* × Shandweel 1	124.1 ± 3.0^a^	19.9 ± 0.5^e^	58.7 ± 3.0^b^	49.11 ± 2.0^ab^	38.97 ± 0.9^b^	Immune
*Yr18* × Gemmiza 11	117.2 ± 4.0^c^	20.2 ± 1.0^d^	50.9 ± 1.0^c^	40.97 ± 1.0^e^	37.19 ± 0.8^b^	Highly resistant
*Yr10*	118.2 ± 3.0^c^	22.5 ± 0.8^c^	42.2 ± 1.0^e^	50.11 ± 3.0^a^	48.25 ± 1.0^a^	Immune
*Yr18*	124.2 ± 4.0^a^	28.2 ± 1.0^a^	63.3 ± 2.0^a^	48.12 ± 3.0^b^	49.11 ± 1.0^a^	Immune
Gemmiza11	110.7 ± 3.0^f^	16.5 ± 0.7^g^	19.1 ± 1.0^i^	30.39 ± 1.0^h^	24.29 ± 0.5^e^	Highly susceptible
Shandweel 1	115.1 ± 3.0^d^	19.9 ± 0.8^e^	36.6 ± 1.0^f^	31.75 ± 1.0^gh^	22.11 ± 0.5^e^	Susceptible
BC1	123.2 ± 3.0^ab^	21.5 ± 1.0^d^	46.0 ± 2.0^d^	41.40 ± 2.0^de^	28.23 ± 0.4^d^	Highly resistant
BC2	112.2 ± 3.0^e^	19.2 ± 0.7^e^	32.1 ± 3.0^g^	38.31 ± 2.0^f^	26.22 ± 0.6^d^	Susceptible
BC3	115.1 ± 2.0^d^	17.0 ± 0.8^g^	53.4 ± 3.0^c^	40.60 ± 2.0^e^	28.29 ± 0.6^d^	Highly resistant
BC4	113.2 ± 2.0^e^	18.1 ± 0.8^f^	24.6 ± 2.0^h^	32.60 ± 1.0^g^	26.0 ± 0.7^d^	Susceptible

Mean values of three replicates and standard deviation (± SD) in each column followed by a different lower-case letter are significantly different according to Duncan’s multiple range tests at *p* ≤ 0.05

Specifically, the susceptible cultivars (Gemmiza 11 and Shandweel 1) and backcrosses (BC2 and BC4) recorded the lowest values for plant height (110.7, 115.1, 112.2, and 113.5 cm), number of tillers (16.5, 19.9, 19.2, and 18.1), number of green spikes (19.1, 36.6, 32.1, and 24.6), 1000-seed weight (30.39, 31.75, 38.31, and 32.6 g), and grain yield (24.29, 22.11, 26.22, and 26.0 g), respectively. Notably, Yr18 recorded the maximum plant height (124.2 cm), number of tillers (28.2), number of green spikes (63.3), 1000-seed weight (48.1 g), and grain yield (49.1 g).

### Evaluation of non-enzymatic antioxidants

The data in Table 5 showed that total phenolic and flavonoid contents varied significantly among wheat cultivars depending on their resistance or susceptibility to yellow rust. Under infection, the total phenolic content in leaves ranged from 3.5 to 5.8 mg/g. The highest values were recorded in the highly resistant cultivars, while the lowest were observed in susceptible and highly susceptible cultivars. Similarly, flavonoid content ranged from 3.1 to 8.0 mg/g. The highly resistant cultivars (*Yr10*,* Yr18 Yr18* × Shandweel 1, and *Yr10* × Shandweel 1) showed the maximum flavonoid levels, whereas the highly susceptible cultivar Gemmiza 11 exhibited the lowest.

**Table 5:** Effect of yellow rust on non-enzymatic antioxidants and free radical scavenging activities in leaves of wheat cultivars and crosses

Cultivars and crosses	Phenolic (mg gallic acid/g)	Flavonoids (mg Catechin/g)	DPPH %	FRAP activity (mg/g)	Reducing Power (µg/g)	ABTS (mg TE/g)	Superoxide free radical scavenging activity (%)	Hydroxyl radical scavenging activity (%)	Nitric Oxide free radical scavenging activity (%)
*Yr18* × Shandweel 1	5.7 ± 0.02^a^	6.7 ± 0.2^c^	55.2 ± 0.8^b^	17.6 ± 0.1^a^	172.2 ± 1.5^b^	2.5 ± 0.01^c^	72.1 ± 0.8^a^	40.5 ± 0.5^b^	70.2 ± 1.0^b^
*Yr10* × Gemmiza 11	5.2 ± 0.01^b^	6.0 ± 0.1^d^	45.6 ± 0.9^c^	15.2 ± 0.1^d^	160.5 ± 1.6^c^	2.4 ± 0.01^cd^	65.3 ± 0.9^c^	36.7 ± 0.4^c^	69.1 ± 1.1^bc^
*Yr10* × Shandweel 1	5.8 ± 0.03^a^	7.5 ± 0.2^b^	53.4 ± 1.0^b^	16.2 ± 0.1^bc^	177.6 ± 2.0^b^	2.6 ± 0.01^bc^	70.5 ± 0.8^b^	42.3 ± 0.4^ab^	75.3 ± 1.3^a^
*Yr18* × Gemmiza 11	5.2 ± 0.012^b^	5.8 ± 0.1^d^	43.8 ± 0.9^cd^	15.9 ± 0.1^c^	155.9 ± 1.4^cd^	2.3 ± 0.01^d^	63.4 ± 0.7^cd^	35.9 ± 0.8^cd^	70.1 ± 0.9^b^
*Yr10*	5.7 ± 0.02^a^	7.8 ± 0.3^ab^	60.1 ± 1.2^a^	17.0 ± 0.2^ab^	180.1 ± 2.2^ab^	2.7 ± 0.02^b^	72.3 ± 0.7^a^	43.9 ± 0.6^a^	77.2 ± 1.2^a^
*Yr18*	5.6 ± 0.02^a^	8.0 ± 0.^3a^	58.9 ± 1.1^a^	16.8 ± 0.1^b^	185.3 ± 2.0^a^	2.9 ± 0.02^a^	74.5 ± 0.8^a^	44.0 ± 0.7^a^	75.8 ± 1.3^a^
Gemmiza11	3.7 ± 0.01^e^	3.1 ± 0.^1h^	22.8 ± 0.4^g^	9.8 ± 0.1^g^	103.5 ± 1.2^g^	1.5 ± 0.01^f^	47.8 ± 0.5^f^	20.1 ± 0.3^g^	45.3 ± 0.7^f^
Shandweel 1	4.0 ± 0.01^d^	4.6 ± 0.1^fg^	35.3 ± 0.5^e^	12.0 ± 0.1^f^	135.2 ± 1.1^e^	1.8 ± 0.01^e^	55.6 ± 0.5^e^	28.5 ± 0.3^f^	59.5 ± 0.8^d^
BC1	4.8 ± 0.01^c^	5.9 ± 0.2^de^	42.5 ± 0.7^d^	14.8 ± 0.2^de^	157.8 ± 1.4^c^	2.2 ± 0.02^d^	60.5 ± 0.6^d^	33.4 ± 0.4^de^	67.8 ± 0.9^c^
BC2	4.0 ± 0.01^d^	4.8 ± 0.1^f^	33.2 ± 0.4^ef^	12.1 ± 0.1^f^	130.4 ± 1.2^ef^	1.7 ± 0.01^e^	53.8 ± 0.6^e^	26.5 ± 0.4^f^	57.6 ± 0.6^de^
BC3	4.9 ± 0.01^c^	5.5 ± 0.2^e^	44.7 ± 0.4^c^	14.3 ± 0.3^e^	152.3 ± 1.6^d^	2.3 ± 0.02^d^	62.9 ± 0.6^d^	31.6 ± 0.5^e^	65.8 ± 0.7^c^
BC4	3.5 ± 0.01^e^	4.3 ± 0.1^g^	31.9 ± 0.3^f^	12.5 ± 0.2^f^	128.7 ± 1.0^f^	1.7 ± 0.01^e^	53.9 ± 0.4^e^	27.6 ± 0.2^f^	55.3 ± 0.8^e^
5%	0.2	0.35	2.76	0.57	5.85	0.11	2.05	1.82	2.27

Mean values of three replicates and standard deviation (± SD) in each column followed by a different lower-case letter are significantly different according to Duncan’s multiple range tests at *p* ≤ 0.05

### Evaluation of free radical scavenging activities

The data in Table 5 showed that a higher DPPH free radical scavenging activity (%) indicated stronger scavenging capacity against the DPPH radical. The highly resistant cultivars (*Yr10*,* Yr18 Yr18* × Shandweel 1, and *Yr10* × Shandweel 1) exhibited the highest DPPH activity, while the highly susceptible cultivar Gemmiza 11 showed the lowest. Similarly, FRAP and reducing power assays revealed maximum activity in immune and highly resistant cultivars and minimum activity in susceptible and highly susceptible ones.

In addition, ABTS reducing power was greatest in the highly resistant cultivars (Yr10, Yr18, Yr18 × Shandweel 1, and Yr10 × Shandweel 1). Furthermore, superoxide anion radical, hydroxyl radical, and nitric oxide radical scavenging activities were consistently higher in the highly resistant cultivars (*Yr10* × Gemmiza 11, *Yr18* × Gemmiza 11, BC1, and BC3) compared with the susceptible (Shandweel 1, BC2, and BC4) and highly susceptible (Gemmiza 11) cultivars.

### Molecular marker analysis

**Fig. 2:**
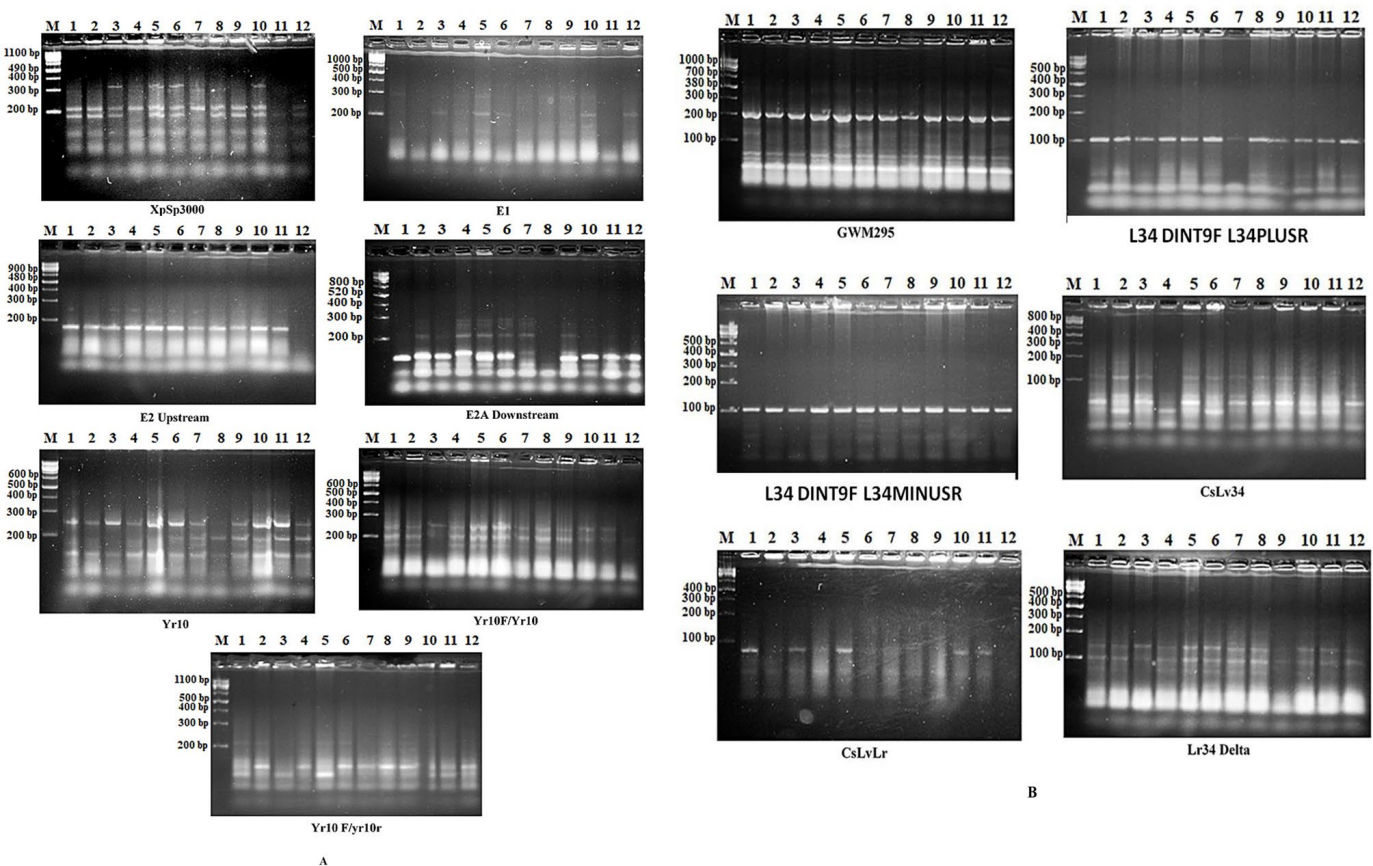
Profiling of gene Yr10-SSR primers on Egyptian wheat cultivars, two Yr monogenic lines (Yr10 and Yr18), crosses, and backcrosses (A), and profiling of gene Yr18-SSR primers on Egyptian wheat cultivars, two Yr monogenic lines (Yr10 and Yr18), crosses, and backcrosses (B), where (1) Yr18 × Shandweel 1, (2) Yr10 × Gemmiza 11, (3) Yr10 × Shandweel 1, (4) Yr18 × Gemmiza 11, (5) Yr10, (6) Yr18, (7) Gemmiza 11, (8) Shandweel 1, (9) BC1, (10) BC2, (11) BC3, (12) BC4

In the present study, to determine the yellow rust resistance genes in two Egyptian wheat cultivars, two Yr monogenic lines (*Yr10* and *Yr18*), crosses and backcrosses, thirteen SSR primers were used. SSR primers were applied to two Egyptian wheat cultivars, two Yr monogenic lines (*Yr10* and *Yr18*), crosses, and backcrosses to determine the genetic diversity. Data from the results of the thirteen tested primers is given in Fig. 2 (a & b).

A total of 82 bands were produced, out of which 33 were monomorphic, 49 were polymorphic, and 7 were unique, as shown in Tables 6 and 7. The CsLvLr primer gave the highest percentage of polymorphism (100%), while the GWM295 primer gave the lowest percentage of polymorphism (30%). In addition, the E2A downstream primer recorded the highest number of polymorphic bands (6), while the Lr34 Delta primer generated the lowest number (2). The E2 upstream primer provided the highest number of unique bands/primer (3).

**Table 6:** SSR analysis of wheat cultivars and crosses using thirteen primers

Marker	Yr18 × Shandweel 1	Yr10 × Gemmiza 11	Yr10 × Shandweel 1	Yr18 × Gemmiza 11	Yr10	Yr18	Gemmiza11	Shandweel 1	BC1	BC2	BC3	BC4
Primer XpSp3000												
200	1	1	1	1	1	1	1	1	1	1	1	1
300	1	1	1	1	1	1	1	1	1	1	1	1
400	1	1	1	1	1	1	1	1	1	1	1	1
490	1	1	1	0	1	1	0	1	1	1	1	1
510	1	1	1	1	1	1	1	1	1	1	0	1
1000	1	1	0	1	1	1	1	0	0	0	0	0
1100	0	0	1	0	1	1	0	0	0	1	0	0
Total	**6**	**6**	**6**	**5**	**7**	**7**	**5**	**5**	**5**	**6**	**4**	**5**
Primer E1												
200	1	1	1	1	1	1	1	1	1	1	1	1
300	1	0	1	1	1	0	1	1	1	1	0	1
400	0	0	0	0	1	0	0	0	1	1	0	0
500	0	0	1	0	1	1	0	0	0	1	0	1
1000	1	0	0	1	1	0	1	1	1	0	0	0
Total	**3**	**1**	**3**	**3**	**5**	**2**	**3**	**3**	**4**	**4**	**1**	**2**
Primer E2 upstream												
200	1	1	1	1	1	1	1	1	1	1	1	1
300	1	1	1	1	1	1	1	1	1	1	1	0
400	1	1	1	1	1	1	1	1	1	1	1	0
480	1	1	1	1	1	1	1	1	1	1	1	0
900	1	0	1	1	1	1	1	0	1	1	1	0
Total	**5**	**4**	**5**	**5**	**5**	**5**	**5**	**4**	**5**	**5**	**5**	**1**
Primer E2A downstream												
100	1	1	1	1	1	1	1	1	1	1	1	1
200	1	1	1	1	1	1	1	1	1	1	1	1
300	0	1	1	1	1	1	1	1	1	1	1	1
400	1	1	1	0	1	1	0	0	1	1	1	1
410	0	0	0	1	0	0	0	0	0	0	0	0
520	0	1	0	1	1	1	1	0	1	0	0	0
800	0	1	0	1	1	1	1	0	1	0	0	0
1000	0	0	0	1	1	1	1	0	0	0	0	0
Total	**3**	**6**	**4**	**7**	**7**	**7**	**6**	**3**	**6**	**4**	**4**	**4**
Primer Yr10												
200	1	1	1	1	1	1	1	1	1	1	1	1
300	1	1	1	1	1	1	1	1	1	1	1	1
400	1	1	1	1	1	1	1	1	1	1	1	1
410	1	1	0	1	1	0	1	0	0	1	0	0
500	0	0	0	0	1	1	1	1	1	1	1	1
600	1	1	1	1	1	1	1	0	1	1	1	1
Total	**5**	**5**	**4**	**5**	**6**	**5**	**6**	**4**	**5**	**6**	**5**	**5**
Primer Yr10F/Yr10												
200	1	1	1	1	1	1	1	1	1	1	1	1
300	1	1	1	1	1	1	1	1	1	1	1	1
400	1	1	0	1	1	1	0	1	1	0	0	0
410	1	1	0	1	1	1	1	1	1	1	1	0
500	1	1	1	1	1	1	1	1	1	1	1	0
600	1	1	1	1	1	1	1	1	1	1	1	1
Total	**6**	**6**	**4**	**6**	**6**	**6**	**5**	**6**	**6**	**5**	**5**	**3**
Primer Yr10 F/yr10r1												
100	1	1	1	1	1	1	1	1	1	1	1	1
200	1	1	1	0	1	1	0	1	1	1	1	1
300	1	1	0	1	0	1	1	1	1	1	1	1
400	1	1	0	0	1	1	1	1	0	0	0	0
1100	1	1	0	1	1	1	0	0	1	0	0	0
Total	**5**	**5**	**2**	**3**	**4**	**5**	**3**	**4**	**4**	**3**	**3**	**3**
Primer GWM295												
100	1	1	1	1	1	1	1	1	1	1	1	1
150	1	1	1	1	1	1	1	1	1	1	1	1
200	1	1	1	1	1	1	1	1	1	1	1	1
250	1	1	1	1	1	1	1	1	1	1	1	1
300	1	1	1	1	1	1	1	1	1	1	1	1
350	1	0	0	1	1	1	1	1	1	1	1	1
380	1	0	0	1	1	1	1	1	1	1	0	1
700	1	1	1	1	1	1	1	1	1	1	1	1
1000	1	1	1	1	1	1	1	1	1	1	1	1
2000	1	0	1	1	1	1	1	0	1	1	1	1
Total	**10**	**7**	**8**	**10**	**10**	**10**	**10**	**9**	**10**	**10**	**9**	**10**
Primer L34 DINT9F L34PLUSR												
100	1	1	1	1	1	1	1	1	1	1	1	1
200	1	1	1	1	1	1	1	1	1	1	1	1
250	0	1	0	1	1	1	0	1	0	1	1	1
300	1	1	0	1	1	1	0	1	1	1	1	1
350	1	1	0	1	1	0	0	1	0	1	0	1
400	0	0	0	1	1	0	0	0	0	0	0	1
500	1	1	1	1	1	1	1	1	1	1	1	1
Total	**5**	**6**	**3**	**7**	**7**	**5**	**3**	**6**	**4**	**5**	**4**	**7**
Primer L34 DINT9F L34MINUSR												
100	1	1	1	1	1	1	1	1	1	1	1	1
200	1	1	0	1	0	0	1	1	1	1	1	1
250	1	1	0	1	1	1	1	1	1	1	1	1
300	1	0	0	0	0	1	0	1	0	0	0	0
400	1	0	0	1	0	1	0	1	0	0	0	0
500	1	1	1	1	1	1	1	1	1	1	1	1
Total	**6**	**4**	**2**	**4**	**3**	**5**	**4**	**6**	**4**	**4**	**4**	**4**
Primer CsLv34												
100	1	1	1	1	1	1	1	1	1	1	1	1
150	1	1	1	1	1	1	0	1	1	1	1	0
200	1	1	1	1	1	1	1	1	1	1	1	1
300	1	1	1	1	1	1	1	1	1	1	1	1
400	1	1	1	1	1	1	1	1	1	1	1	1
500	0	0	0	0	1	1	0	1	1	1	1	1
600	1	1	1	0	1	1	1	1	1	1	1	1
700	0	1	0	0	1	1	0	0	0	1	0	0
800	0	1	0	0	0	0	0	0	0	0	0	0
Total	**6**	**8**	**6**	**5**	**8**	**8**	**5**	**7**	**7**	**8**	**7**	**6**
Primer CsLvLr												
100	1	1	1	1	1	1	1	0	1	0	1	0
300	1	1	1	1	1	1	1	1	1	1	1	0
400	1	0	1	0	1	0	0	0	0	1	1	0
Total	**3**	**2**	**3**	**2**	**3**	**2**	**2**	**1**	**2**	**2**	**1**	**0**
Primer Lr34 Delta												
100	1	1	1	1	1	1	1	1	1	1	1	1
200	1	1	1	1	1	1	1	1	1	1	1	1
400	1	0	0	1	1	1	1	1	0	1	1	1
500	1	1	1	1	1	1	1	1	1	1	1	1
600	1	1	1	1	1	1	1	1	1	1	1	1
650	1	0	0	0	1	1	1	1	0	0	0	0
Total	**6**	**4**	**4**	**5**	**6**	**6**	**6**	**6**	**4**	**5**	**5**	**5**

**Table 7:** Bands variation and polymorphism percentage for wheat cultivars and crosses using thirteen SSR primers

Primer	Total No. of bands	Monomorphic bands	Polymorphic bands	Unique bands	Band size	Cultivars	Polymorphism%
XpSp3000	7	3	4	1	510	BC3	57
E1	5	1	4	0			80
E2 upstream	5	1	4	3	300,400,480	BC4	80
E2A downstream	8	2	6	2	300, 410	*Yr18* × Shandweel 1 *Yr18* × Gemmiza 11	75
Yr10	6	3	3	1	600	Shandweel 1	50
Yr10F/Yr10	6	3	3	1	500	BC4	60
Yr10 F/yr10r	5	1	4	0			80
GWM295	10	7	3	0			30
L34 DINT	7	3	4	0			57
L34 DINT 9	6	2	4	0			67
CsLv34	9	4	5	1	600	*Yr18* × Gemmiza 11	56
CsLvLr	3	0	3	1	300	BC4	100
Lr34 Delta	6	4	2	0			33
Total No. of bands	**83**	**43**	**49**	**10**			**59.8**

The E2 upstream primer revealed the highest number of unique bands—three negative markers—in BC4 (susceptible to yellow rust), with molecular sizes of 300, 400, and 480 bp. The E2 downstream primer produced two unique bands: a negative marker (300 bp) in the immune genotype Yr18 × Shandweel 1, and a positive marker (410 bp) in *Yr18* × Gemmiza 11, which is highly resistant. The XpSp3000 primer amplified a single negative marker (510 bp) in BC3, a highly resistant genotype. For the Yr10 primer, a negative marker (600 bp) was detected in Shandweel 1 (susceptible), while the Yr10F/Yr10 primer revealed another negative marker (500 bp) in BC4 (susceptible). The CsLv34 primer detected a negative marker (600 bp) in *Yr18* × Gemmiza 11 (highly resistant), whereas the CsLvLr primer produced a negative marker (300 bp) in BC4 (susceptible).

### UPGMA cluster analysis

**Fig. 3:**
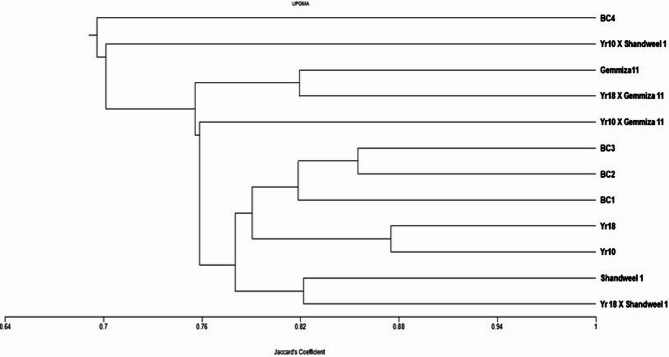
UPGMA cluster analysis based on the Jaccard similarity coefficient, showing the genetic relationships among the twelve wheat cultivars and crosses tested, obtained from SSR primers

The dendrogram of the wheat cultivars and crosses, constructed using thirteen SSR markers, is shown in Fig. 3. Based on UPGMA cluster analysis, the genotypes were grouped into two main clusters. The first cluster contained BC4, which exhibited a distinct phenetic lineage and pronounced susceptibility to yellow rust disease. The second cluster comprised the remaining 11 cultivars and crosses, further divided into two sub-clusters. The first sub-cluster included the cross *Yr10* × Shandweel 1, while the second sub-cluster grouped the other 10 cultivars and crosses, reflecting their high level of genetic similarity. This clustering pattern underscores the genetic divergence of BC4, consistent with its pronounced susceptibility, while the tight grouping of the other genotypes reflects their shared genetic basis for resistance.

Table (8) presents the similarity matrix among the 12 wheat cultivars and crosses analyzed using SSR markers. The highest similarity (87.5%) was observed between the two Yr monogenic lines (*Yr10* and *Yr18*), both of which are immune to yellow rust disease. In contrast, the lowest similarity (62.7%) was recorded between the cross *Yr10* × Shandweel 1 and *Yr18* × Gemmiza 1. This pattern suggests that high genetic similarity may be associated with shared resistance genes, whereas greater divergence could reflect the presence of different resistance mechanisms.

**Table 8:** Similarity matrix values generated using NTSYS software for the data produced from thirteen SSR primers for the wheat cultivars and crosses

	Yr 18 X Shandweel 1	Yr10 X Gemmiza 11	Yr10 X Shandweel 1	Yr18 X Gemmiza 11	Yr10	Yr18	Gemmiza11	Shandweel 1	BC1	BC2	BC3	BC4
*Yr18* × Shandweel 1	1.000											
*Yr10* × Gemmiza 11	0.773	1.000										
*Yr10* × Shandweel 1	0.708	0.686	1.000									
*Yr18* ×Gemmiza 11	0.803	0.760	0.627	1.000								
*Yr10*	0.802	0.763	0.701	0.790	1.000							
*Yr18*	0.797	0.779	0.693	0.763	0.875	1.000						
Gemmiza11	0.784	0.716	0.671	0.819	0.772	0.766	1.000					
Shandweel 1	0.822	0.730	0.639	0.737	0.741	0.779	0.740	1.000				
BC1	0.800	0.781	0.739	0.787	0.810	0.805	0.792	0.781	1.000			
BC2	0.799	0.737	0.768	0.722	0.835	0.785	0.724	0.784	0.811	1.000		
BC3	0.767	0.746	0.781	0.707	0.734	0.773	0.732	0.771	0.826	0.855	1.000	
BC4	0.667	0.644	0.667	0.653	0.684	0.675	0.653	0.739	0.718	0.797	0.758	1.000

### Multivariate heatmap

**Fig. 4:**
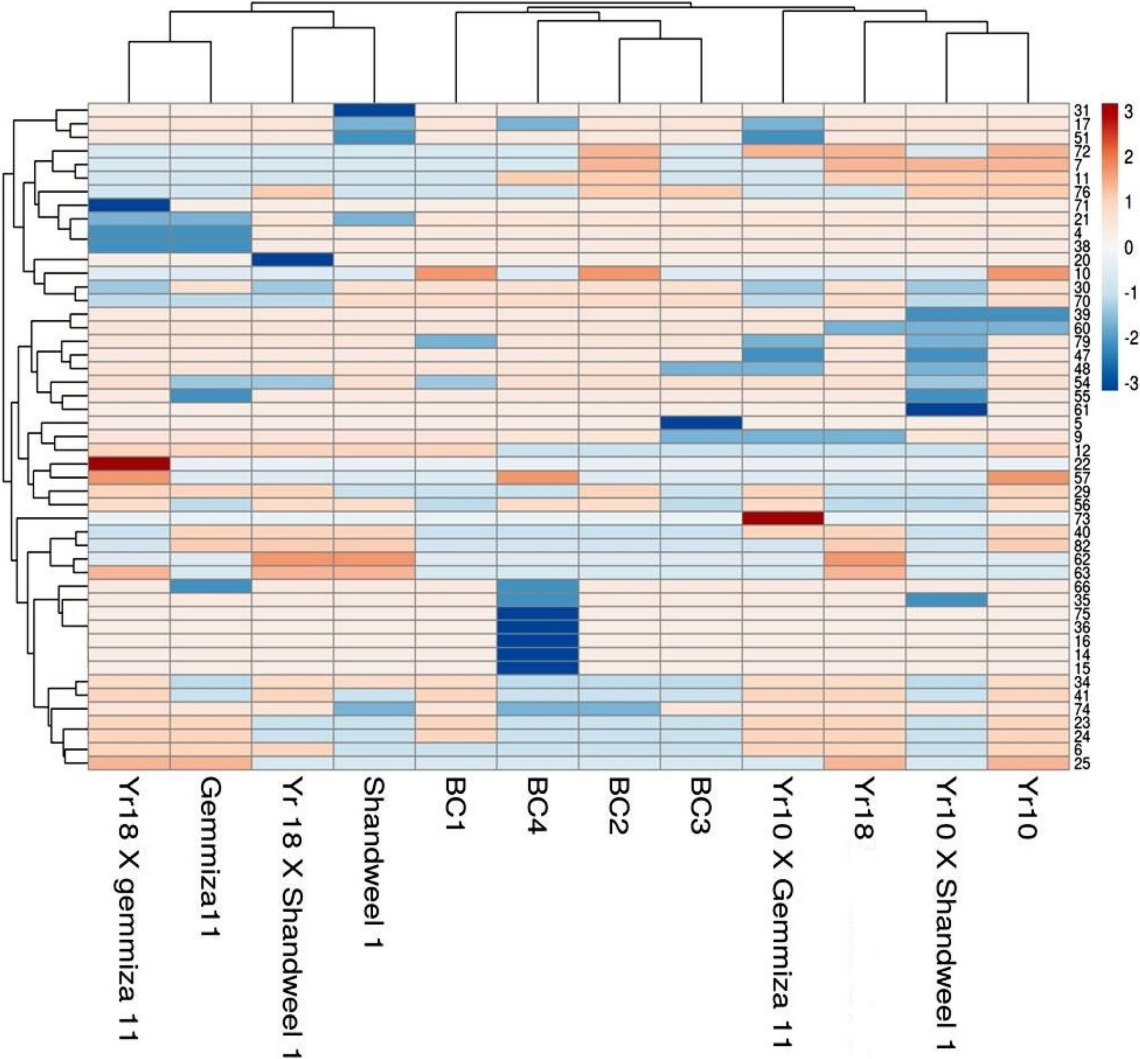
Multivariate Heatmap illustrating the genetic diversity of wheat cultivars and their crosses, based on thirteen SSR primers for using the module of a Heatmap of ClustVis—an online tool for clustering and visualizing of multivariate data

A heatmap provides detailed information about the genetic variation seen in different plant breeds, and multivariate compound similarity analysis is typically used to extract more information about this genetic variation. A heatmap generated by the web tool ClustVis, which groups and shows compound similarities in multivariate data. The three clusters that resulted from the wheat cultivars and crosses are shown in the columns (Fig. 4). The first cluster was divided into four sub-clusters: (*Yr10*), (*Yr10* × Shandweel1), (*Yr18*), and (*Yr10* × Gemmiza11). The second cluster was divided into four sub-clusters: (BC1), (BC2), (BC3), and (BC4). In addition, the third cluster was split into four sub-clusters: (Shandweel 1), (*Yr18* × Shandweel 1), (Gemmiza 11), and (*Yr18* × Gemmiza 11).

### Principal component analysis (PCA)

**Fig. 5:**
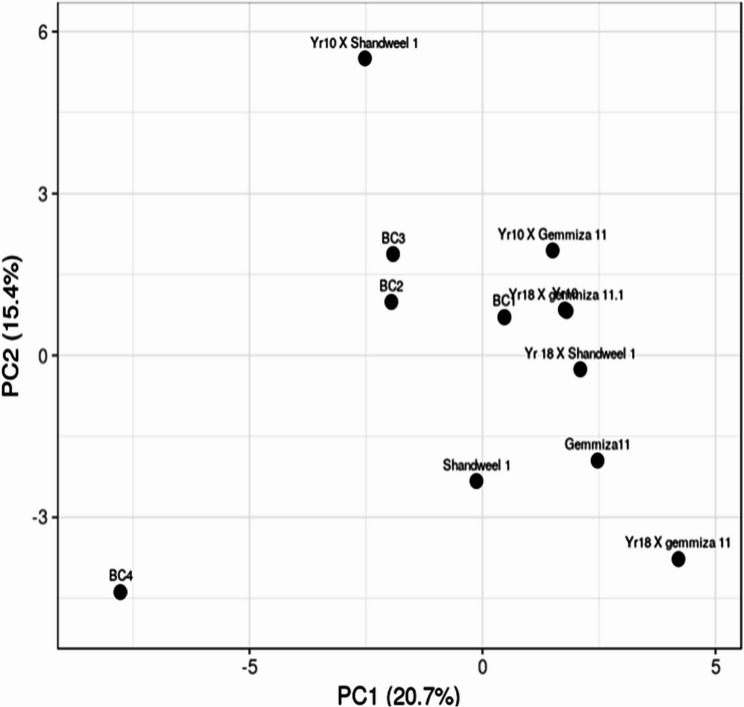
An illustration of the genetic diversity expressed in wheat cultivars and their crosses, according to a principal component analysis (PCA) based on polymorphism of thirteen SSR markers, using PAST software

The genetic diversity of the investigated genotypes was evaluated using SSR marker data, multivariate clustering, and PCA analysis. The PCA scatter plot demonstrated the effectiveness of SSR markers in distinguishing the tested genotypes. Two principal components were identified: PCA1 (20.7%) and PCA2 (15.4%). The analysis clearly separated the yellow rust–susceptible genotype into a distinct group, while the remaining genotypes clustered closely together in a single group (Fig. 5).

## Discussion

One of the most damaging diseases that drastically reduces production worldwide is stripe rust disease of wheat, which can reduce yields by up to 70% when sensitive cultivars are cultivated in the field [46]. A more ideal approach to lessen the damage caused by stripe rust is to utilize resistant cultivars, as the agronomic and chemical ways of controlling the disease are costly [47]. Throughout all of the world’s wheat-growing regions, tolerance to yellow (stripe) rust is one of the most crucial goals of breeding programs [48]. To create new and varied resistant germplasm, wheat landraces from various geographical areas may provide unique rust resistance genes [49].

In the present study, Gemmiza 11 and Shandweel 1 were highly susceptible wheat cultivars and showed lower plant growth and yield attributes. On the other hand, the two Yr monogenic lines (*Yr10* and *Yr18*) showed high resistance against stripe rust. Furthermore, a cross between the monogenic lines of resistance and the susceptible cultivars showed immune-related and extremely resistant responses to *P. striiformis*. Moreover, the back crosses vary in their susceptibility and resistance to pathogens. BC1 and BC3 showed high resistance to wheat strip rust, while BC2 and BC4 showed susceptibility to wheat strip rust. Wheat production is negatively impacted by rust diseases because the fungus severely damages the susceptible host plant’s vascular system, which restricts the flow of water and nutrients from the soil to the growing kernel and other organs [51–54]. Shrunken grains result from this interfering with photosynthate transfer [50]. Similar results have been presented by Elshafei et al. [51]. Highly susceptible cultivars always have very little endosperm formation and entirely shriveled grains.

Although infected plants respond to pathogens through diverse morphological, biochemical, and molecular mechanisms, their primary defense is the rapid generation of ROS, which leads to oxidative stress [55]. To counteract ROS, both enzymatic and non-enzymatic antioxidant systems play a critical role [56]. In particular, low redox potential phenols are important for the cyclic reduction of ROS [57]. In the present study, the immune and highly resistant cultivars exhibited the highest concentrations of phenolics and flavonoids, whereas the highly susceptible cultivars had the lowest levels. These findings are consistent with those of Singla et al. [56], who reported that resistant barley cultivars infected with *P. striiformis* f. sp. hordei accumulated greater amounts of phenols and flavonoids. Phenolic content is known to increase under biotic stress, such as insect feeding or pathogen infection, and phenolic compounds significantly contribute to the induced resistance phenotype [58]. Flavonoids and their derivatives further enhance plant protection, as observed in sorghum [59]. Following infection with *Burkholderia andropogonis*, several flavonoid-related compounds were upregulated, some of which act as phytoalexins through sustained accumulation, thereby limiting pathogen growth and reproduction [60].

Free radical scavenging activity in the present study differed significantly among cultivars, reflecting their varying levels of resistance or susceptibility to yellow rust disease. Notably, all immune wheat cultivars showed enhanced scavenging activity against superoxide anion radicals, hydroxyl radicals, and nitric oxide radicals. A positive correlation was observed between total phenols and flavonoids and antioxidant potential, as measured by DPPH free radical scavenging activity, FRAP, reducing power, and ABTS assays. This suggests that higher accumulation of phenolic compounds is associated with greater antioxidant capacity to neutralize diverse free radicals generated under stress conditions [56]. Similar findings for FRAP and DPPH activities were reported in chickpea plants, where infestation by Helicoverpa armigera increased antioxidant activity in leaves, pod walls, and developing seeds [61]. The reducing power of phenolic compounds is often attributed to their ability to transform Fe³⁺ to Fe²⁺ ions [25]. In wheat, *Pyricularia oryzae* infection has been shown to induce a stronger antioxidative response in moderately resistant genotypes compared to susceptible ones [62]. Furthermore, nitric oxide appears to function as a signaling molecule in activating defense-related pathways [63].

A total of 82 bands were generated using 13 SSR primers in the current investigation; of these, 33 were monomorphic, 49 were polymorphic, and 7 were unique. The highest percentage of polymorphism (100%) was recorded with the CsLvLr primer, whereas the lowest percentage (30%) was observed with the GWM295 primer. Furthermore, the Lr34Δ primer produced the fewest polymorphic bands (2), while the E2A downstream primer generated the highest number of polymorphic bands (6). The greatest number of unique bands per primer (3) was detected with the E2 upstream primer. Mahmoud et al. [48] found that bulked segregant analysis (BSA) combined with three molecular marker systems, simple sequence repeats (SSR), sequence-related amplified polymorphism (SRAP), and random amplified polymorphic DNA (RAPD), successfully differentiated resistant cultivars from susceptible ones. SSR markers demonstrated the highest polymorphism (43.6%), making them more informative, reproducible, and reliable in detecting genetic variation. This makes SSRs a powerful tool for marker-assisted selection in wheat breeding programs. Since molecular markers linked to target genes enable the identification of desirable traits without field testing, they improve breeding efficiency, particularly for traits difficult to evaluate or strongly influenced by the environment. Therefore, identifying new yellow rust resistance genes and developing associated molecular markers is essential for their effective incorporation and pyramiding into wheat cultivars [64].

Yuan et al. [65] used tagged markers to investigate the frequency of *Yr* genes among wheat genotypes. Similarly, Zeng et al. [66] analyzed 330 elite cultivars and 164 advanced breeding lines in China, detecting *Yr9*,* Yr17*,* Yr18*, and *Yr26* in 134 (29.4%), 45 (9.1%), 10 (2%), and 15 (3%) entries, respectively. Yan et al. [67] evaluated twenty-five cultivars against stripe rust in the field from 2014 to 2018, alongside eleven molecular markers linked to known *Yr* genes, and reported the presence of *Yr1*,* Yr13*,* Yr18*,* Yr14a*,* Yr26*,* Yr34*, and *Yr46*, either individually or in combination. Notably, Yr5 has been demonstrated to be effective against all North American virulent rust races [68]. In India, Haider et al. [69] identified thirteen *Yr* gene-associated markers in resistant wheat germplasm, with 38 genotypes showing high resistance and seven showing moderate resistance. Among these, *Yr5*,* Yr10*,* Yr15*, and *Yr24/26* were the most frequent, being detected in 16, 10, 14, and 15 lines, respectively. Taken together, these international findings underscore the global importance of diverse *Yr* genes in breeding programs and highlight that combining multiple resistance loci, such as those identified in the present study, can provide durable protection against stripe rust.

## Conclusion

The crosses between resistant cultivars (*Yr10*,* Yr18*) and susceptible ones (Gemmiza 11, Shandweel 1), along with their backcrosses, enhanced genetic resistance to yellow rust and carried multiple resistance genes. The resistant cultivars demonstrated stronger antioxidant defense systems alongside gene-based resistance, underscoring their value in wheat improvement programs. Rather than emphasizing individual antioxidant assays (FRAP, DPPH, reducing power), the overall pattern highlights the role of biochemical and molecular traits in enhancing disease resistance. The presence of slow-rusting resistance genes was confirmed by phenotypic responses. Marker-assisted selection (MAS) can be used to combine effective resistance genes, supporting Egypt’s wheat breeding programs in developing durable rust-resistant cultivars.

## Data Availability

All data generated or analyzed during this study are included in this published article.
